# Insulin-Like Growth Factor II mRNA-Binding Protein 3 Expression Correlates with Poor Prognosis in Acral Lentiginous Melanoma

**DOI:** 10.1371/journal.pone.0147431

**Published:** 2016-01-21

**Authors:** Yi-Shuan Sheen, Yi-Hua Liao, Ming-Hsien Lin, Hsien-Ching Chiu, Shiou-Hwa Jee, Jau-Yu Liau, Yih-Leong Chang, Chia-Yu Chu

**Affiliations:** 1 Department of Dermatology, National Taiwan University Hospital and National Taiwan University College of Medicine, Taipei, Taiwan; 2 Graduate Institute of Pathology, National Taiwan University College of Medicine, Taipei, Taiwan; 3 Graduate Institute of Clinical Medicine, National Taiwan University College of Medicine, Taipei, Taiwan; 4 Department of Surgery, National Taiwan University Hospital Hsin-Chu Branch, Hisn-Chu, Taiwan; 5 Department of Pathology, National Taiwan University Hospital, Taipei, Taiwan; University of Alabama at Birmingham, UNITED STATES

## Abstract

Insulin-like growth factor-II mRNA-binding protein 3 (IMP-3) is an RNA-binding protein expressed in multiple cancers, including melanomas. However, the expression of IMP-3 has not been investigated in acral lentiginous melanoma (ALM). This study sought to elucidate its prognostic value in ALMs. IMP-3 expression was studied in 93 patients diagnosed with ALM via immunohistochemistry. Univariate and multivariate analyses for survival were performed, according to clinical and histologic parameters, using the Cox proportional hazard model. Survival curves were graphed using the Kaplan-Meier method. IMP-3 was over-expressed in 70 out of 93 tumors (75.3%). IMP-3 expression correlated with thick and high-stage tumor and predicted poorer overall, melanoma-specific, recurrence-free and distant metastasis-free survivals (*P* = 0.002, 0.006, 0.008 and 0.012, respectively). Further analysis showed that patients with tumor thickness ≤ 4.0 mm and positive IMP-3 expression had a significantly worse melanoma-specific survival than those without IMP-3 expression (*P* = 0.048). IMP-3 (hazard ratio 3.67, 95% confidence intervals 1.35–9.97, *P* = 0.011) was confirmed to be an independent prognostic factor for melanoma-specific survival in multivariate survival analysis. Positive IMP-3 expression was an important prognostic factor for ALMs.

## Introduction

Insulin-like growth factor II mRNA-binding protein 3 (IMP-3) is expressed in fetal tissues in a time-dependent and cell-dependent manner, but it is undetectable in human adult tissues [1, 2]. IMP-3 appears to play an important role in the differentiation process of embryogenesis [3]. Moreover, IMP-3 was shown to promote proliferation of human leukemia cells and invasion of hepatocellular carcinoma [1, 3]. Evidence from an *in vitro* IMP-3 knockdown research and from IMP-3 administration in lung cancer patients illustrated that IMP-3 may be a therapeutic target for malignancies [1]. Increased IMP-3 expression was described in carcinomas of the colon, kidney and liver, as well as in melanomas [3–7].

Pryor et al. found that IMP-3 was expressed in melanomas but not in benign nevi, even when dysplastic features were present and IMP-3 was expressed in metastatic melanomas significantly more than in thin melanomas [4]. Yu et al. noted higher and more diffuse IMP-3 staining in deep melanomas compared with superficial ones [5]. In our previous study, IMP-3 expression was much stronger in advanced-stage/metastatic melanomas and correlated with poor overall survival (OS) [8]. However, the association of IMP-3 and melanoma-specific survival (MSS) had not yet been defined in acral lentiginous melanomas (ALMs). In this study, we correlated IMP-3 expression with clinicopathological parameters as well as prognosis to further dissect the role of IMP-3 in a larger cohort of ALMs. In addition, IMP-3 expression was positively associated with high mobility group AT-hook 2 (HMGA2) expression in melanoma [8]. HMGA2 overexpression was demonstrated to be associated with *BRAF/NRAS* mutations [8, 9]. *KIT* mutations were frequently found in acral melanomas [10]. Therefore, we also investigated the relationship between IMP-3 expression and *BRAF/NRAS/KIT* mutation status in this study.

## Materials and Methods

### Patients and tissues

This retrospective cohort study was approved by the Research Ethics Committee of National Taiwan University Hospital (NTUH-REC No.: 201303064RIND) and it was conducted according to the principles of the Declaration of Helsinki. The cohort was constructed from paraffin-embedded, formalin-fixed tissue blocks which were obtained from the National Taiwan University Hospital Department of Pathology archives. Cases without adequate histologic material and medical history data were excluded. All patients who enrolled in the study provided written informed consent to use their resected tissues and received standard-of-care treatment, i.e., a wide excision of the primary lesion [8]. A total of 132 biopsies, including 3 dysplastic nevi, 13 benign nevi, 7 ALMs in situ, 93 ALMs, and 16 metastatic ALMs, were enrolled. Sixty-seven ALMs were reported in our previous study [8]. The specimens between January 1995 and July 2013 were resected, with a follow-up range between 2 to 20 years and with a median follow-up time of 51.5 months. Clinical data describing patient demographics, clinical course, and follow-up through April 1, 2015 were obtained from the medical record and the Cancer Registry, Medical Information Management Office, National Taiwan University Hospital. Survival rates for 93 patients with stages I to IV ALMs were calculated. Melanoma-specific survival was defined as time from the initial melanoma diagnosis and considered censored for patients who were alive at last follow-up or who died without evidence of melanoma [11]. Overall survival was defined as time from diagnosis to death from any cause or last follow-up. Recurrence-free survival (RFS) was defined as the length of time after treatment during which no local recurrence, regional, or distant metastasis was found. Distant metastasis-free survival (DMFS) was defined as the length of time after treatment during which no distant metastases was found. The following clinical variables were considered: age (<65 versus ≥65 years), sex (male versus female), Breslow thickness (stratified by ≤1, 1.01–2, 2.01–4 or >4 mm), ulceration (absent versus present), location (upper versus lower extremity), metastatic nodes at diagnosis (absent versus present) and American Joint Committee on Cancer (AJCC) stage (stratified by I, II, III or IV) [11, 12].

### Immunohistochemical analysis

Tissue sections were collected from formalin-fixed and paraffin-embedded primary tumors [8]. Immunohistochemical studies were performed on 5-μm formalin-fixed, paraffin-embedded tissue sections using a mouse monoclonal antibody against L523S/IMP-3 (1:100; Epitomics, Burlingame, CA, USA). Tissue sections were deparaffinized according to established procedures. Sections were washed in PBST. Antigen retrieval was performed using target retrieval solution, modified citrate buffer, pH6.0, in a steamer at 121°C for 15 minutes. Slides were rinsed with PBST for 5 minutes and quenched with 0.3% H_2_O_2_ for 15 minutes. After blocking, sections were incubated with the monoclonal mouse anti-L523/IMP-3 antibody at 4°C overnight. The sections were then incubated for 30 minutes with MultiLink antibody (BioGenex, Fremont CA, USA) for 40 minutes. This was followed by incubation with the streptavidin peroxidase (BioGenex) for 20 minutes. Staining was developed with 3-amino-9-ethylcarbazole (AEC) (BioGenex) for 10 minutes. Slides were rinsed in running PBST, counterstained with modified Mayer’s hematoxylin, and blued in 0.3% ammonia water followed by a tap water rinse. After mounting, slides were viewed using a Nikon Eclipse E600 light microscope equipped with an Olympus DP70 digital camera. Cytoplasmic staining was regarded as positive for IMP-3 expression. For negative control, we substituted the primary antibody with 5% fetal bovine serum. The proportion of tumor cells positive for IMP-3 was recorded as diffuse positive (>50%), focal or heterogeneous (10–50%), and trace (<10%) according to previous reports [3–5, 13, 14]. The intensity of IMP-3 staining was graded as negative, weak, moderate or strong according to previous reports [4, 5, 14]. Representative sections with immunohistochemical staining intensity against IMP-3 are provided in S1 Fig. Melanoma was classified as 0 if melanin was either absent or present in <5% of cells, 1 if melanin was visible in 5% to 50% of cells (moderately pigmented), and 2 if melanin was present in > 50% of cells (strongly pigmented) according to previous report to evaluate the relationship between melanogenesis and outcome [15]. Two independent observers blinded to the clinical data independently evaluated the immunostaining reactions. The interobserver variability was 10%, but, in case of discrepancy, a consensus was formed of three observers.

### Mutation analyses of *BRAF*, *NRAS* and *KIT* genes in melanoma tissues

DNA was isolated from three consecutive 10-μM sections of each formalin-fixed, paraffin-embedded tissue sample. Genomic DNA was extracted using the QIAamp® DNA FFPE Tissue Kit (Qiagen, Hilden, Germany) according to the manufacturer's protocols. DNA concentration was quantified using an A 260 absorbance with a BioPhotometer (Eppendorf, Hamburg, Germany). Genomic DNA (50–100 ng/sample) was used as a template. The isolated DNA was used for real-time LightCycler PCR (LC PCR) with LightMix® kit *BRAF* V600E, a new assay method for *BRAF* mutation detection, and methylation-specific PCR analyses [8]. For *NRAS* mutation detection, exons 2 and 3 of the *NRAS* gene were amplified by PCR in at least two separate preparations of genomic DNA as described previously [8, 16]. For *KIT* mutation detection, exons 11, 13, 17 and 18 of the *KIT* gene were amplified by PCR as described previously [16]. The PCR primers for exon 9 were 5’-CCTAGAGTAAGCCAGGGCTTT-3’ (forward) and 5’-GACAGAGCCTAAACATCCCCT-3’ (reverse).

### Statistical analysis

The data were summarized by descriptive statistics. Odds ratios, 95% confidence intervals (CI), and *P*-values were calculated using univariate logistic regression. Associations between IMP-3 and *KIT* mutations were assessed with Fisher's exact test. Associations between IMP-3 and melanin were assessed with chi-square test. Survival probabilities were calculated using the Kaplan-Meier method and were examined by log-rank tests [8]. The influence of each variable on survival was assayed using univariate analysis by the Cox proportional hazard models. Age, sex, thickness, ulceration, lymph node metastasis, IMP-3, and upper-extremity location were entered into multivariate survival analysis. Since thickness, ulceration and lymph node metastasis were components of stage, stage was not involved in the multivariate analyses. The assumptions of proportional hazard analyses were verified by supremum tests. All statistical tests were two-sided, and a *P*-value of 0.05 or less was considered to be statistically significant. All statistical analyses were executed using SAS 9.2 (Cary, North Carolina, USA).

## Results

### Expression of IMP-3 protein in ALM

Tissues from a total of 93 patients with primary ALM were analyzed (Table 1). The study included 48 men and 45 women with a mean age of 65 years (median: 66 years; range: 24–89 years). Most tumors (79/93, 84.9%) were located on the lower extremities. Ulceration was present in 36.6% (34/93) of patients. The mean Breslow thickness of tumor was 3.8 mm. At the time of diagnosis, 35.5% of the cases represented stage I, 40.9% were at stage II, 18.3% were at stage III, and only 5.4% were classified as stage IV. Eighty-two patients (88.2%) were followed up for more than 5 years or until death. The median follow-up duration after diagnosis was 4.29 years (range: 0.6–20.2 years). The 5-year MSS rate for all 93 ALM patients was 56.8%.

**Table 1:** Clinical and histologic prognostic factors and IMP-3 expression.

	IMP-3 expression			
Characteristic	Total	≥10% of tumor cells, n (%)	OR (95% CI)	*P*-value
**Age (years)**				
** <65**	**41**	**28 (68.3%)**	**1.00**	
** ≥65**	**52**	**42 (80.8%)**	**1.95 (0.75–5.06)**	**0.170**
**Sex**				
** Female**	**45**	**30 (66.7%)**	**1.00**	
** Male**	**48**	**40 (83.3%)**	**2.50 (0.94–6.66)**	**0.067**
**Ulceration**				
** No**	**59**	**44 (74.6%)**	**1.00**	
** Yes**	**34**	**26 (76.5%)**	**1.11 (0.41–2.97)**	**0.839**
**Tumor thickness (mm)**				
** ≤1**	**18**	**9 (50%)**	**1.00**	
** 1.01–2.00**	**29**	**21 (72.4%)**	**2.63 (0.77–9)**	**0.125**^a^
** 2.01–4.00**	**18**	**16 (88.9%)**	**8 (1.41–45.4)**	**0.019**^a^
** >4**	**28**	**24 (85.7%)**	**6 (1.47–24.45)**	**0.012**^a^
**Lymph node metastasis**				
** No**	**73**	**52 (71.2%)**	**1.00**	
** Yes**	**20**	**18 (90%)**	**3.63 (0.77–17.05)**	**0.102**
***BRAF/NRAS* mutations**^b^				
** No**	**65**	**49 (75.4%)**	**1.00**	
** Yes**	**22**	**19 (86.4%)**	**2.07 (0.54–7.91)**	**0.289**
**Upper-extremity location**				
** No**	**79**	**46 (74.7%)**	**1.00**	
** Yes**	**14**	**11 (78.6%)**	**1.24 (0.32–4.91)**	**0.756**
**AJCC stage at diagnosis**				
** I**	**33**	**19 (57.6%)**	**1.00**	
** II**	**38**	**31 (81.6%)**	**3.26 (1.12–9.53)**	**0.031**^c^
** III**	**17**	**16 (94.1%)**	**11.79 (1.39–99.7)**	**0.024**^c^
** IV**	**5**	**4 (80%)**	**2.95 (0.30–29.32)**	**0.357**^c^

AJCC, American Joint Committee on Cancer; CI, confidence intervals; IMP-3, IGF II mRNA-binding protein 3; OR, odds ratio.

^a^ Comparison of specified group with thickness ≤1 mm.

^b^ Six patients had missing *BRAF/NRAS* mutations data.

^c^ Comparison of specified group with stage I.

**Fig 1:**
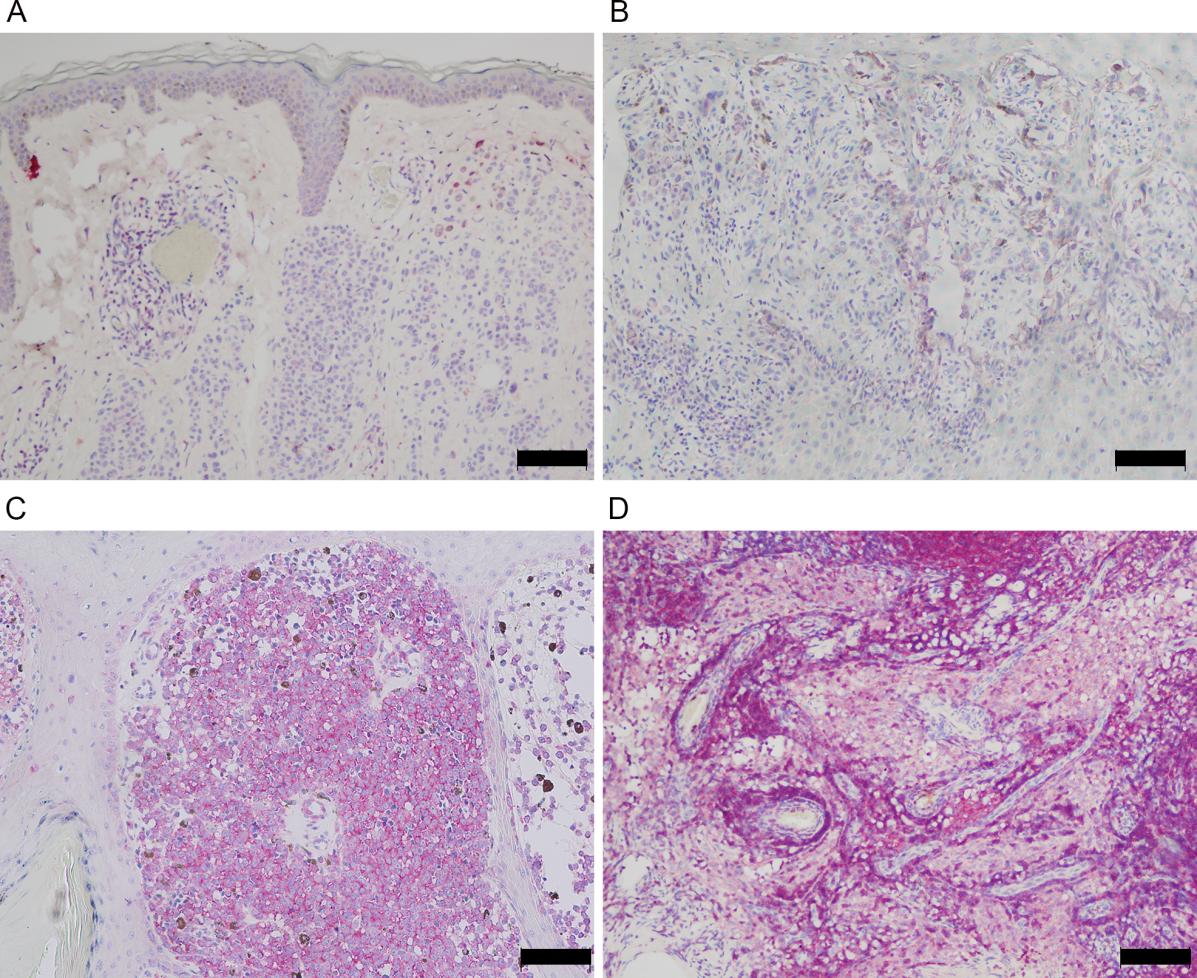
IMP-3 was expressed in ALM and associated with its progression. (**A**) Benign melanocytic nevi such as intradermal nevus were negative for IMP-3. (**B**) Focal IMP-3 expression was found in ALM with thickness ≤ 4 mm (Breslow thickness = 1.2 mm). (**C**) Strong and diffuse cytoplasmic expression was noted in ALM with depth >4.0mm (Breslow thickness = 6 mm). **(D**) Metastatic melanoma expressed IMP-3 in most tumor cells (Bar, 100 μm).

Benign nevi were negative for IMP-3 (Fig 1A). In 70.8% (46/65) of melanomas with thickness ≤ 4 mm, IMP-3 positivity was detected in at least 10% of the tumor cells (Table 1 and Fig 1B). 85.7% (24/28) of melanomas with depth >4.0mm expressed IMP-3 in at least 10% of the tumor cells (Table 1 and Fig 1C). No staining was seen in the nontumorous tissues adjacent to the tumors (Fig 1C). All 16 metastatic melanomas were positive for IMP-3 (Fig 1D).

In our pilot analysis, MSS in patients with <10% IMP-3 expression was the best (*P* = 0.008; log rank test), significantly better than for patients with >50% or 10–50% IMP-3 expression (*P* = 0.003 and 0.035; log-rank test). The MSS between patients with >50% and 10–50% IMP-3 expression was similar (*P* = 0.207, log-rank test). Therefore, we dichotomized IMP-3 staining data as positive (≥10% of cancer cells stained positive) or negative (<10% of cancer cells stained positive) for further survival analysis.

### Clinicopathological and prognostic significance of IMP-3 expression

Patients with tumors recorded as having thickness of 2.01–4.0 mm or >4.0 mm had significantly more IMP-3 expression than those with thickness of ≤1.0 mm (Table 1, *P* = 0.019 and 0.012, respectively). IMP-3 expression had no significant difference between the 1.01–2.0 mm and ≤1.0 mm thickness groups. IMP-3 expression was more likely to be high in stage II (*P* = 0.031) and III (*P* = 0.024) than in stage I melanomas. IMP-3 expression did not correlate with age, sex, ulceration, metastatic nodes or tumor site in ALMs. *NRAS* and *BRAF* mutations appeared in only 13.8% (12/87) and 8% (7/87) of patients, respectively; and IMP-3 expression was not correlated with *NRAS/BRAF* mutations (Table 1). We screened a subset of 29 ALMs (23 tumors were IMP-3 positive) for *KIT* mutations and identified 3 IMP-3-positive tumors which were *KIT*-mutated. IMP-3 overexpression was not associated with *KIT* mutations (*P* = 1; Fisher’s exact test) in this subset of the study population.

**Fig 2:**
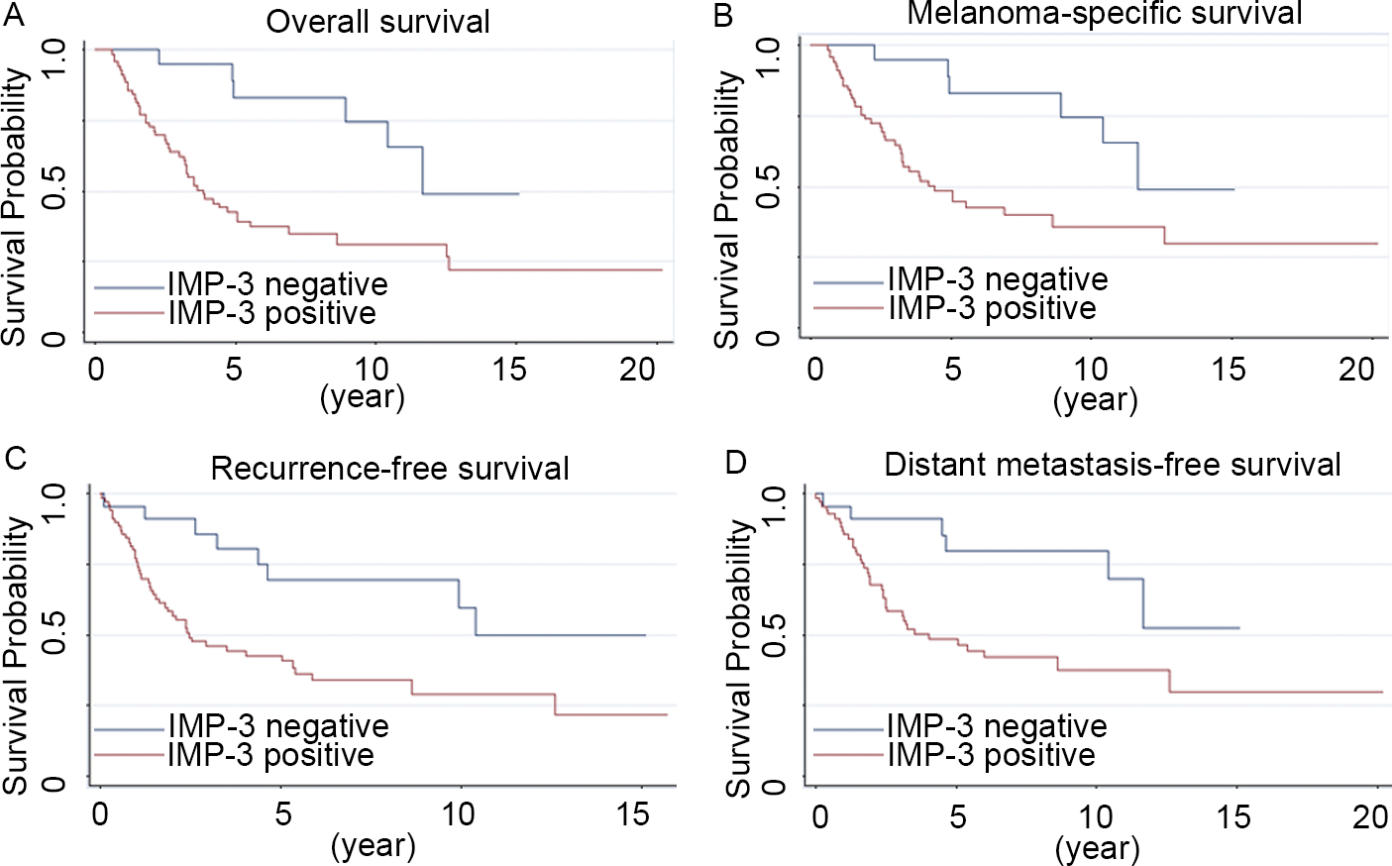
Kaplan-Meier curves of survival associated with IMP-3 expression in 93 primary ALMs. IMP-3 overexpression was significantly associated with overall (**A**), melanoma-specific (**B**), recurrence-free (**C**), and distant metastasis-free survival (**D**) (*P* = 0.002, 0.006, 0.008 and 0.012, respectively; log rank test).

Patients with positive IMP-3 expression had significantly worse OS and MSS than those with negative IMP-3 expression (Fig 2A and 2B; *P* = 0.002 and 0.006, respectively; log rank test). Stratified by positive (n = 70) and negative IMP-3 expression (n = 23), the 5-year MSS rates were 48.7% and 83.1%, respectively. The 5-year RFS rate for IMP-3 positive patients was 42.7%, whereas for those with negative IMP-3 expression was 69.7% (Fig 2C; *P* = 0.008; log rank test). IMP-3 overexpression was also significantly associated with DMFS with a 5-year DMFS rate of 47.8% in IMP-3 positive versus 79.7% in the IMP-3-negative patients (Fig 2D; *P* = 0.012; log rank test).

### Concomitant effects of IMP-3 expression with thickness on the prognosis of ALM

**Fig 3:**
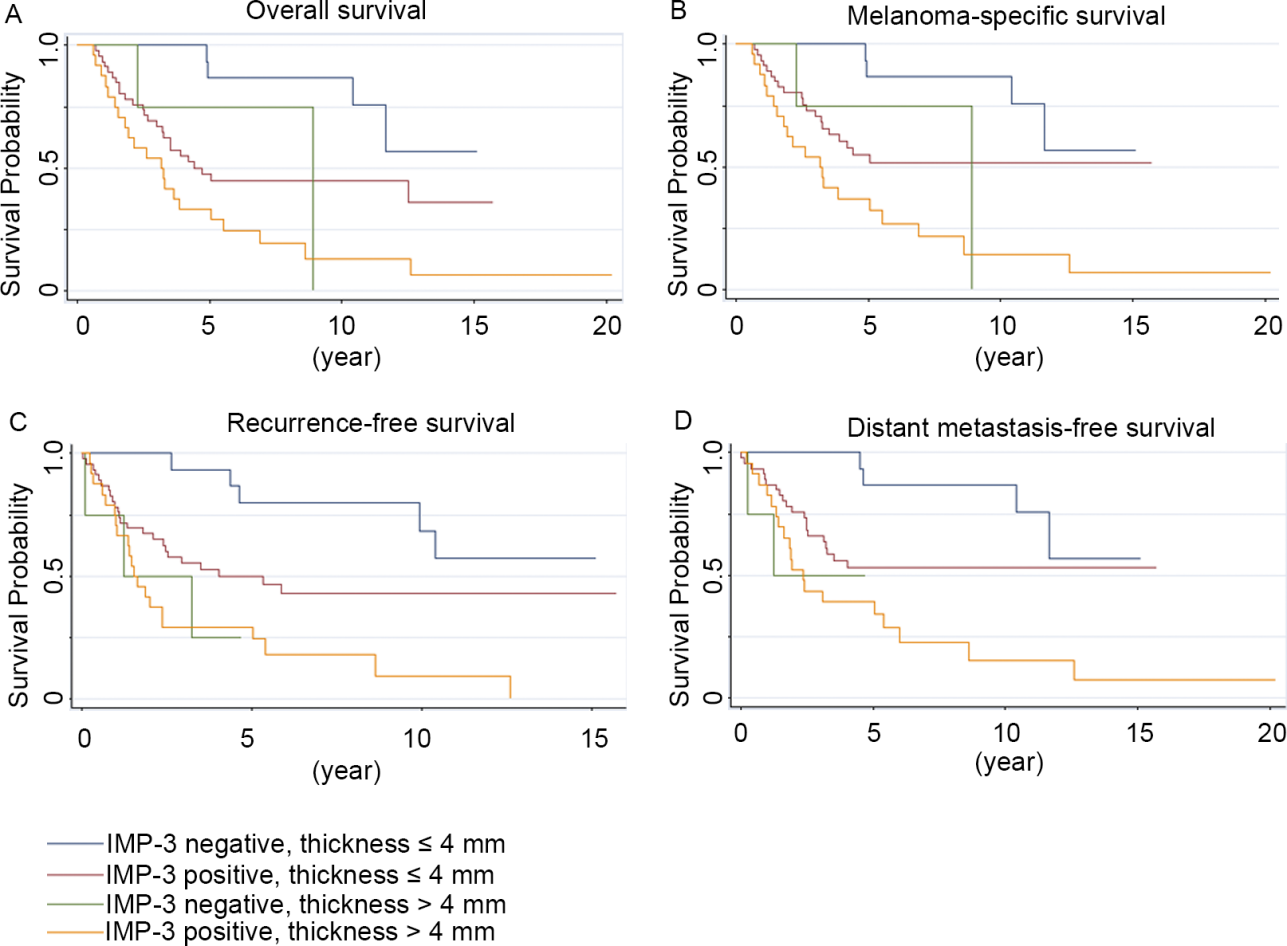
Kaplan-Meier analysis of survival in patients with or without IMP-3 expression in relation to thickness. The *P* value was obtained from comparison of four groups (log rank test). Patient with tumor thickness ≤ 4.0 mm and negative IMP-3 expression had the best overall (**A**), melanoma-specific (**B**), recurrence-free (**C**), and distant metastasis-free survival (**D**) (*P*<0.001, *P*<0.001, *P*<0.001, and *P* = 0.001, respectively).

Patient with tumor thickness ≤ 4.0 mm and negative IMP-3 expression had the best OS, MSS, RFS and DMFS (Fig 3A–3D; *P*<0.001, *P*<0.001, *P*<0.001, and *P* = 0.001, respectively; log rank test). To determine the effect of IMP-3 expression on the prognosis in tumors measuring 4.0 mm or less, we performed a combination analysis. Those patients with tumor thickness ≤ 4.0 mm concomitant with positive IMP-3 expression had poorer OS, MSS, RFS and DMFS than ALM patients with thickness ≤ 4 mm and negative IMP-3 expression (Fig 3A–3D; *P* = 0.012, 0.048, 0.026 and 0.045, respectively; log rank test). Patients with tumors thicker than 4.0 mm had worse OS, MSS, RFS and DMFS regardless of the expression status of IMP-3 (*P* = 0.227, 0.266, 0.942 and 0.934 respectively; log rank test).

### IMP-3 expression is an independent prognostic factor in ALM

Thickness >4.0 mm (HR, 2.65; 95% CI, 1.12–6.26; *P* = 0.026), metastatic nodes (HR, 5.19; 95% CI, 2.8–9.64; *P*<0.001), AJCC stage III (HR, 7.65; 95% CI, 3.24–18.04; *P*<0.001) and IV (HR, 9.41; 95% CI, 3.09–28.59; *P*<0.001), and IMP-3 (HR, 3.17; 95% CI, 1.34–7.52; *P* = 0.009) were associated with MSS in univariate analysis (Table 2). Univariate analysis showed that male (hazard ratio (HR), 1,78; 95% CI, 1.02–3.11; *P* = 0.044), thickness >4.0 mm (HR, 2.45; 95% CI, 1.09–5.53; *P* = 0.031), lymph node metastasis (HR, 4.33; 95% CI, 2.39–7.86; *P*<0.001), AJCC stage III (HR, 6.25; 95% CI, 2.8–13.99; *P*<0.001) and IV (HR, 8.03; 95% CI 2.74–23.49; *P*<0.001), and IMP-3 (HR, 3.64; 95% CI, 1.55–8.58; *P* = 0.003) expression predicted an unfavorable OS (S1 Table). As shown in Table 2 and S1 Table, multivariate survival analysis found that IMP-3 proved to be an independent prognostic factor for MSS (HR, 3.67; 95% CI, 1.35–9.97; *P* = 0.011) and OS (HR, 3.84; 95% CI, 1.46–10.12; *P* = 0.006). Lymph node status remained the most decisive prognostic factor for predicting MSS (HR, 6.37; 95% CI, 3.16–12.89; *P*< 0.001) and OS (HR, 5.11; 95% CI, 2.62–9.96; *P*< 0.001).

**Table 2:** Univariate and multivariate analysis of risk factors associated with melanoma-specific survival (MSS) in acral lentiginous melanoma patients.

Variable	Univariate HR (95% CI)	Univariate P-value	Multivariate HR (95% CI)^c^	Multivariate P-value
**Age, ≥65**	**1.03 (0.58–1.84)**	**0.509**	**1.33 (0.67–2.63)**	**0.416**
**Sex, male**	**1.73 (0.95–3.13)**	**0.072**	**1.89 (0.97–3.68)**	**0.063**
**Tumor thickness, mm**				
** ≤1.00**^a^	**1.00**		**1.00**	**-**
** 1.01–2.00**	**1.06 (0.41–2.76)**	**0.901**	**0.62 (0.22–1.73)**	**0.363**
** 2.01–4.00**	**0.83 (0.28–2.47)**	**0.731**	**0.27 (0.08–0.89)**	**0.031**
** >4.00**	**2.65 (1.12–6.26)**	**0.026**	**1.21 (0.45–3.23)**	**0.707**
**Ulceration**	**1.48 (0.82–2.67)**	**0.192**	**1.60 (0.85–3.03)**	**0.148**
**Lymph node metastasis**	**5.19 (2.80–9.64)**	**<0.0001**	**6.37 (3.16–12.89)**	**<0.0001**
**Stage**^b^				
** I**^a^	**1.00**		**-**	**-**
** II**	**1.61 (0.72–3.62)**	**0.247**	**-**	**-**
** III**	**7.65 (3.24–18.04)**	**<0.0001**	**-**	**-**
** IV**	**9.41 (3.09–28.59)**	**<0.0001**	**-**	**-**
**IMP-3**	**3.17 (1.34–7.52)**	**0.009**	**3.67 (1.35–9.97)**	**0.011**
**Upper-extremity location**	**1.30 (0.63–2.70)**	**0.483**	**0.95 (0.41–2.17)**	**0.895**

AJCC, American Joint Committee on Cancer; CI, confidence intervals; IMP-3, IGF II mRNA-binding protein 3;HR, hazard ratio.

^a^Reference.

^b^Since thickness, ulceration and lymph node metastasis were components of stage, stage was not involved in the multivariate analyses.

^c^Age, sex, thickness, ulceration, lymph node metastasis, IMP-3 and upper-extremity location were entered into multivariate survival analysis.

With regard to RFS and DMFS, IMP-3 was not the independent prognostic factor in the multivariate analysis (S2 and S3 Tables). Male gender (HR, 2.66; 95% CI, 1.42–4.97; *P* = 0.002) and metastatic nodes (HR, 8.84; 95% CI, 4.25–18.36; *P*<0.001) were independent risk factors that were associated with RFS. Only lymph node status was an independent risk factor associated with worse DMFS (HR, 7.55; 95% CI, 3.55–16.08; *P*<0.001).

## Discussion

Amongst skin cancers, cutaneous melanoma is the most frequent cause of mortality in Caucasian populations, with incidence rates per 100,000 patient years ranging between 33.5 in Americans and to 59.1 among Australian males [17–21]. However, the incidence of melanoma in Asia is significantly lower, with incidence rates of 0.2 to 0.5 per 100,000 patient years [22, 23]. The most common histological subtype of melanoma among Asian populations is ALM [24–27], which accounts for 63.3% of all cutaneous melanoma cases among Chinese [28, 29], 50% among Singaporeans [17], 54.5% among Koreans [10] and 40.5% among Japanese [30]. However, ALM constitutes only 3–10% of all melanoma cases among Caucasians [25, 27, 31]. Because of their typically inconspicuous locations on the body, ALMs are not usually noticed until the late stages, when they are ulcerated or large in size, so they tend to have a grave prognosis [17, 32, 33]. Hence, the establishment of more molecular markers for the prediction of invasiveness and metastatic potential will aid in the development of better strategies for patient management.

Pryor et al. suggested that IMP-3 might represent a prognostic marker in melanoma but they were unable to show that patients with thin melanomas that expressed IMP-3 had more aggressive behavior [4]. In concordance with their finding, Yu et al. also noted stronger and more diffuse IMP-3 staining in deep melanomas than in superficial melanomas [5]. Although both studies suggested that IMP-3 expression increased with greater depth and stage, their results did not attain statistical significance due to small sample size [4, 5]. In addition, these studies did not include survival analysis [4, 5]. Recently we reported IMP-3 expression was significantly associated with ALM as compared to other melanoma subtypes and was predictive of poor OS [8]. In the present study, we included long-term MSS, OS, RFS, and DMFS data and we demonstrated IMP-3 overexpression was significantly associated with MSS, OS, RFS and DMFS in ALMs.

We found that IMP-3 protein was significantly associated with tumor thickness and stage II-III (Table 1). Because of small numbers of patients in this subgroup, Stage IV ALM did not have significantly more frequent IMP-3 expression as compared with stage I ALM. Breslow tumor thickness is an important prognostic factor of survival [34]. Interestingly, tumor thickness of 2.01–4.0 mm had a protective effect on MSS as compared to the ≤ 1.0 mm thickness group (Table 2), which could be partly explained by the fact that the tumors were sufficiently large to attract patients’ attention before they metastasized. Although ALM patients with tumor thickness of ≤ 4.0 mm had a relatively good prognosis, a small subset experienced recurrence and death as a result of their melanoma. IMP-3 expression could further discriminate the survival of patients with tumors thickness of ≤ 4.0 mm and it may be of value to identify at-risk patients who might benefit from more aggressive treatment (Fig 3).

Metastatic nodes were the most important independent risk factor for predicting DFS and DMFS in this series, suggesting a virulent biology in the late stage ALMs with aggressive recurrence and metastatic patterns [35]. IMP-3 was not an independent prognostic factor in DFS and DMFS in this series. One possible explanation is that the prognostic significance of the nodal status overpowers the importance of IMP-3. Another explanation is that this sample of 93 patients was relatively small and thus may be insufficiently powerful to detect any differences in DFS and DMFS between IMP-3-positive and -negative patients.

IMP-3 was an independent indicator of poor MSS and OS, which may be explained by the increased invasive potential of IMP-3-expressing tumors [8]. Other reports have found similar results to the effect that IMP-3 could predict a negative prognosis in multiple cancers [3, 6, 7]. MeWo cells with overexpression of IMP-3 demonstrated increased proliferation and migration and significantly enhanced tumorigenesis and metastatic potential in nude mice [8]. It was therefore proposed that IMP-3 played a crucial role in establishing melanomas’ invasive and metastatic ability and hence poor prognosis. It is also worth noting that Jeng et al. discovered that all HMGA2-positive hepatocellular carcinomas were IMP-3 positive, which implied that IMP-3 might be an essential element in the regulation of HMGA2 [3]. HMGA2 played key roles in melanoma progression and prognosis [8, 9]. We discovered that IMP-3 expression was positively associated with HMGA2 expression in melanoma cells and tissues [8]. IMP-3 bound to and stabilized HMGA2 mRNA and improved the migration and invasion of melanoma cells by regulating HMGA2 expression [8]. Furthermore, IMP-3 ribonucleoproteins were demonstrated to operate as cytoplasmic safe houses that kept HMGA2 mRNA from miRNA-directed decay during tumor progression [8, 36]. Taken together, these results suggest that the expression of IMP-3, in addition to causing the direct function of IMP-3 itself, may work in concert with HMGA2 to activate cell migration and invasion programs, thus inducing the highly malignant phenotype seen in clinical samples. HMGA2 overexpression was demonstrated to be associated with *BRAF*/*NRAS* mutations [8, 9]. In addition, *KIT* mutations were frequently found in ALMs [10]. Hence, we investigated the relationship between IMP-3 expression and these mutations. However, IMP-3 expression was not correlated with NRAS/BRAF/KIT mutations in ALMs in this study. IMP-3 staining intensity was also analyzed in these melanoma tissues. Nonetheless, there was no significant difference in the MSS rate between patients with different IMP-3 staining intensities (*P* = 0.435, log-rank test). Melanogenesis was reported to be associated with poorer survival in patients with advanced melanomas [15, 37–39]. Because none of our ALMs was amelanotic, we only analyzed moderately and strongly pigmented melanomas. In 71% (66/93) of melanomas, melanin was detected in 5% to 50% of melanoma cells and 29% (27/93) of melanomas expressed melanin in >50% of cells. Comparing MSS in these two groups in all stages did not reveal significant difference (*P* = 0.694, log-rank test). If only stage 3 and 4 melanomas were compared, no significant difference in MSS was found (*P* = 0.869, log-rank test). Twenty-one of 27 (77.8%) strongly pigmented melanomas and 49 of 66 (74.2%) moderately pigmented melanomas expressed IMP-3 in 10% or more of tumor cells. IMP-3 overexpression was not associated with the percentage of cells containing melanin (*P* = 0.72, chi-square test) in this study population. We did not find significant differences in IMP-3 expression and MSS between moderately and strongly pigmented ALMs. This discrepancy with previous reports may be due to different proportions of melanoma subtypes in the study population [15].

Although we have amassed one of the largest such cohorts reported to date for this prognostic analysis, a larger cohort may identify additional predictive features of IMP-3 expression in ALM patients. Because these observations were derived from a data set from a single institution in Taiwan, whether these data are generalizable to ALM in other populations needs to be validated in additional cohorts including other Asians, Caucasians or Africans.

## Conclusions

IMP-3 expression in ALM tumor tissues could provide complementary prognostic information, in addition to the major clinicopathological features, which in turn could help to determine the best choice of therapy. Further mechanistic researches are warranted to work out whether IMP-3 is a therapeutic target for new modalities in the therapy of ALMs.

## Supporting Information

S1 Fig Representative sections with immunohistochemical staining intensity against IMP-3. (A) IMP-3 staining was negative. (B) IMP-3 staining shows weak intensity. (C) IMP-3 staining shows moderate intensity. (D) IMP-3 staining shows strong intensity (Bar, 100 μm).
(TIF)

S1 Table Univariate and multivariate analysis of risk factors associated with overall survival (OS) in acral lentiginous melanoma patients. (DOCX)

S2 Table Univariate and multivariate analysis of risk factors associated with recurrence-free survival (RFS) in acral lentiginous melanoma patients. (DOCX)

S3 Table Univariate and multivariate analysis of risk factors associated with distant metastasis-free survival (DMFS) in acral lentiginous melanoma patients. (DOCX)
