# Supplementary figures and images for: Insulin-Like Growth Factor II mRNA-Binding Protein 3 Expression Correlates with Poor Prognosis in Acral Lentiginous Melanoma
Source: PLoS One. 2016 Jan 21;11(1):e0147431. doi: 10.1371/journal.pone.0147431 (PMC4721868; doi:10.1371/journal.pone.0147431)

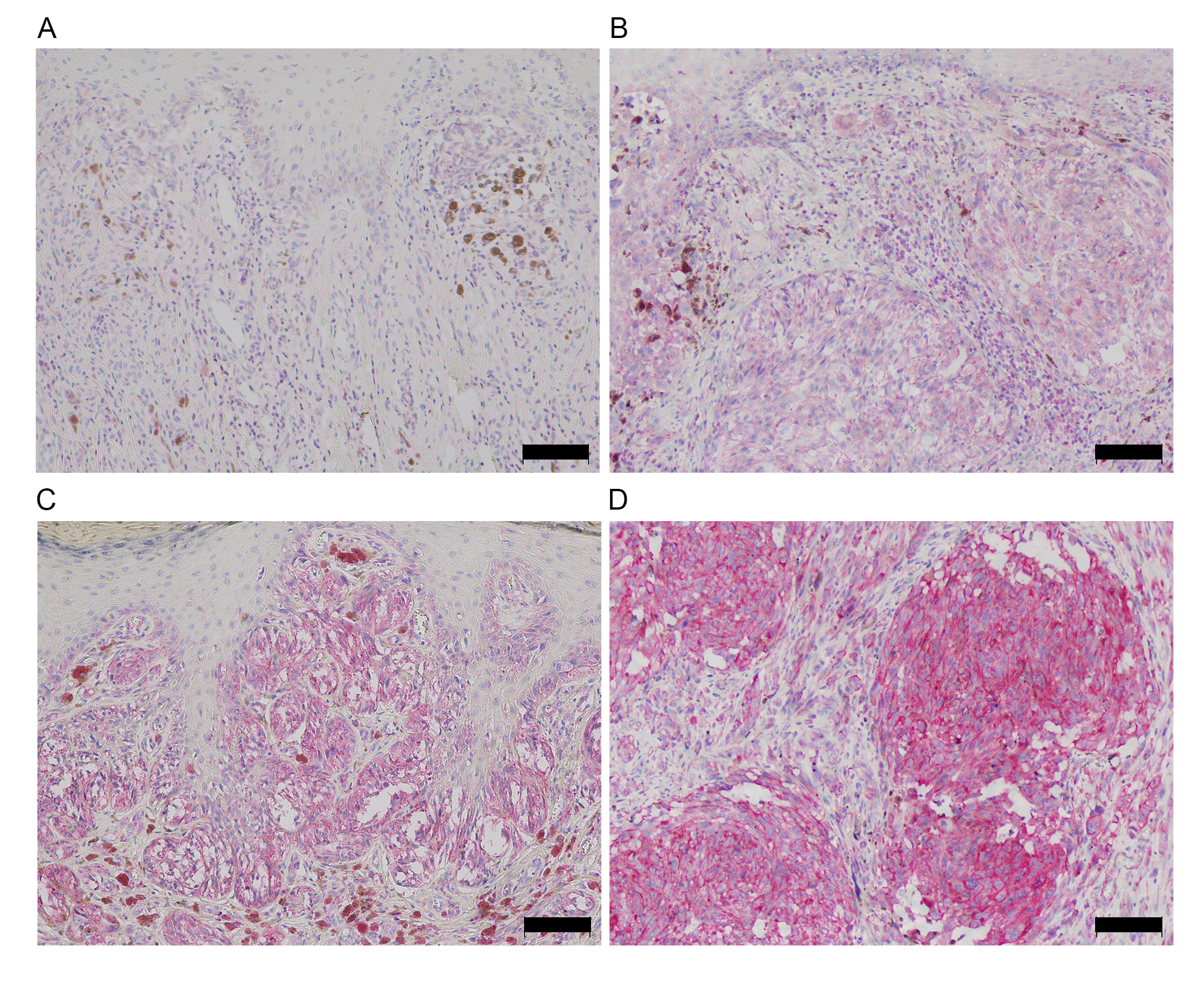

Supplement: S1 Fig — (A) IMP-3 staining was negative. (B) IMP-3 staining shows weak intensity. (C) IMP-3 staining shows moderate intensity. (D) IMP-3 staining shows strong intensity (Bar, 100 μm). (TIF) [file pone.0147431.s001.tif]
