# Supplementary material for: Insulin-Like Growth Factor II mRNA-Binding Protein 3 Expression Correlates with Poor Prognosis in Acral Lentiginous Melanoma
Source: PLoS One. 2016 Jan 21;11(1):e0147431. doi: 10.1371/journal.pone.0147431 (PMC4721868; doi:10.1371/journal.pone.0147431)
Supplement: S1 Table — (DOCX) [file pone.0147431.s002.docx]

| Variable | Univariate HR  (95% CI) | Univariate P-value | Multivariate HR  (95% CI) | Multivariate  P-value |
| --- | --- | --- | --- | --- |
| Age, ≥65 | 1.19 (0.69-2.06) | 0.535 | 1.35 (0.71-2.54) | 0.358 |
| Sex, male | 1.78 (1.02-3.11) | 0.044 | 1.82 (0.98-3.37) | 0.059 |
| Tumor thickness, mm |  |  |  |  |
| ≤1.00^a^ | 1.00 |  | 1.00 | - |
| 1.01-2.00 | 1.03 (0.42-2.54) | 0.951 | 0.63 (0.24-1.65) | 0.342 |
| 2.01-4.00 | 1.08 (0.41-2.80) | 0.879 | 0.37 (0.13-1.08) | 0.068 |
| >4.00 | 2.45 (1.09-5.53) | 0.031 | 1.21 (0.48-3.04) | 0.680 |
| Ulceration | 1.33 (0.76-2.32) | 0.324 | 1.48 (0.81-2.69) | 0.201 |
| Lymph node metastasis | 4.33 (2.39-7.86) | <0.0001 | 5.11 (2.62-9.96) | <0.0001 |
| Stage^b^ |  |  |  |  |
| I^a^ | 1.00 |  | - | - |
| II | 1.62 (0.78-3.36) | 0.199 | - | - |
| III | 6.25 (2.80-13.99) | <0.0001 | - | - |
| IV | 8.03 (2.74-23.49) | 0.0001 | - | - |
| IMP-3 | 3.64 (1.55-8.58) | 0.003 | 3.84 (1.46-10.12) | 0.006 |
| Upper-extremity location | 1.12 (0.54-2.29) | 0.764 | 0.87 (0.39-1.94) | 0.724 |

**S1 Table. Univariate and multivariate analysis of risk factors associated with overall survival (OS) in acral lentiginous melanoma patients.**

*AJCC,* American Joint Committee on Cancer; *CI,* confidence intervals*; IMP-3,* IGF II mRNA-binding protein 3;*HR*, hazard ratio.

^a^Reference.

^b^Since thickness, ulceration and lymph node metastasis were components of stage, stage was not involved in the multivariate analyses.
