# Supplementary material for: Insulin-Like Growth Factor II mRNA-Binding Protein 3 Expression Correlates with Poor Prognosis in Acral Lentiginous Melanoma
Source: PLoS One. 2016 Jan 21;11(1):e0147431. doi: 10.1371/journal.pone.0147431 (PMC4721868; doi:10.1371/journal.pone.0147431)
Supplement: S2 Table — (DOCX) [file pone.0147431.s003.docx]

**S2 Table. Univariate and multivariate analysis of risk factors associated with recurrence-free survival (RFS) in acral lentiginous melanoma patients.**

| **Variable** | **Univariate HR**  **(95% CI)** | **Univariate *P*-value** | **Multivariate HR**  **(95% CI)** | **Multivariate *P*-value** |
| --- | --- | --- | --- | --- |
| Age, ≥65 | 1.07 (0.62-1.84) | 0.816 | 1.61 (0.85-3.04) | 0.144 |
| Sex, male | 1.99 (1.14-3.47) | 0.016 | 2.66 (1.42-4.97) | 0.002 |
| Tumor thickness, mm |  |  |  |  |
| ≤1.00^a^ | 1.00 |  | 1.00 | - |
| 1.01-2.00 | 1.25 (0.52-3.05) | 0.620 | 0.98 (0.37-2.58) | 0.967 |
| 2.01-4.00 | 1.06 (0.40-2.85) | 0.905 | 0.37 (0.11-1.18) | 0.093 |
| >4.00 | 3.24 (1.43-7.33) | 0.005 | 1.61 (0.60-4.32) | 0.345 |
| Ulceration | 1.32 (0.76-2.29) | 0.329 | 1.24 (0.68-2.24) | 0.484 |
| Lymph node metastasis | 6.62 (3.65-12.02) | <0.001 | 8.84 (4.25-18.36) | <0.001 |
| Stage^b^ |  |  |  |  |
| I^a^ | 1.00 |  | - | - |
| II | 1.48 (0.71-3.05) | 0.293 | - | - |
| III | 8.15 (3.74-17.77) | <0.001 | - | - |
| IV | 17.31 (5.63-53.24) | <0.001 | - | - |
| IMP-3 | 2.68 (1.26-5.72) | 0.012 | 1.81 (0.74-4.42) | 0.195 |
| Upper-extremity location | 1.46 (0.73-2.90) | 0.287 | 1.27 (0.57-2.82) | 0.558 |

*AJCC,* American Joint Committee on Cancer; *CI,* confidence intervals*; IMP-3,* IGF II mRNA-binding protein 3;*HR*, hazard ratio.

^a^Reference.

^b^Since thickness, ulceration and lymph node metastasis were components of stage, stage was not involved in the multivariate analyses.
