# Supplementary material for: Insulin-Like Growth Factor II mRNA-Binding Protein 3 Expression Correlates with Poor Prognosis in Acral Lentiginous Melanoma
Source: PLoS One. 2016 Jan 21;11(1):e0147431. doi: 10.1371/journal.pone.0147431 (PMC4721868; doi:10.1371/journal.pone.0147431)
Supplement: S3 Table — (DOCX) [file pone.0147431.s004.docx]

**S3 Table. Univariate and multivariate analysis of risk factors associated with distant metastasis-free survival (DMFS) in acral lentiginous melanoma patients.**

| **Variable** | **Univariate HR**  **(95% CI)** | **Univariate *P*-value** | **Multivariate HR**  **(95% CI)** | **Multivariate *P*-value** |
| --- | --- | --- | --- | --- |
| Age, ≥65 | 0.95 (0.53-1.70) | 0.860 | 1.24 (0.64-2.39) | 0.522 |
| Sex, male | 1.71 (0.95-3.11) | 0.074 | 1.91 (0.1-3.65) | 0.050 |
| Tumor thickness, mm |  |  |  |  |
| ≤1.00^a^ | 1.00 |  | 1.00 | - |
| 1.01-2.00 | 1.10 (0.42-2.84) | 0.852 | 0.774 (0.28-2.16) | 0.625 |
| 2.01-4.00 | 0.86 (0.29-2.57) | 0.786 | 0.37 (0.11-1.23) | 0.106 |
| >4.00 | 2.95 (1.25-6.96) | 0.013 | 1.39 (0.51-3.77) | 0.52 |
| Ulceration | 1.51 (0.84-2.72) | 0.171 | 1.31 (0.7-2.45) | 0.405 |
| Lymph node metastasis | 7.09 (3.69-13.60) | <0.001 | 7.55 (3.55-16.08) | <0.001 |
| Stage^b^ |  |  | - | - |
| I^a^ | 1.00 |  | - | - |
| II | 1.66 (0.74-3.72) | 0.222 | - | - |
| III | 9.87 (4.11-23.71) | <0.001 | - | - |
| IV | 74.56 (19.34-287.47) | <0.001 |  |  |
| IMP-3 | 2.96 (1.25-7.01) | 0.014 | 2.36 (0.88-6.32) | 0.087 |
| Upper-extremity location | 1.57 (0.75-3.26) | 0.229 | 1.49 (0.66-3.39) | 0.336 |

*AJCC,* American Joint Committee on Cancer; *CI,* confidence intervals*; IMP-3,* IGF II mRNA-binding protein 3;*HR*, hazard ratio.

^a^Reference.

^b^Since thickness, ulceration and lymph node metastasis were components of stage, stage was not involved in the multivariate analyses.
